# Inflammatory Ly-6C^hi^ monocytes play an important role in the development of severe transplant arteriosclerosis in hyperlipidemic recipients

**DOI:** 10.1016/j.atherosclerosis.2012.05.010

**Published:** 2012-08

**Authors:** Alexandru Schiopu, Satish N. Nadig, Ovidiu S. Cotoi, Joanna Hester, Nico van Rooijen, Kathryn J. Wood

**Affiliations:** a Transplantation Research Immunology Group, University of Oxford, Nuffield Department of Surgical Sciences, John Radcliffe Hospital, Oxford, UK; b Department of Cellular and Molecular Biology, University of Medicine and Pharmacy of Targu Mures, Targu Mures, Romania; c Department of Molecular Cell Biology, VUMC, Amsterdam, The Netherlands

**Keywords:** Transplant vasculopathy, Hypercholesterolemia, Inflammation, Monocytes, Macrophages

## Abstract

**Objective:**

Transplant arteriosclerosis (TA) restricts long-term survival of heart transplant recipients. Although the role of monocyte/macrophages is well established in native atherosclerosis, it has been studied to a much lesser extent in TA. Plasma cholesterol is the most important non-immunologic risk factor for development of TA but the underlying mechanisms are largely unknown. We hypothesized that monocyte/macrophages might play an important role in the pathogenesis of TA under hyperlipidemic conditions.

**Methods:**

We studied TA in fully mismatched arterial allografts transplanted into hyperlipidemic ApoE^−/−^ recipients compared to wild-type controls. The recruitment of distinct monocyte populations into the grafts was tracked by *in vivo* labelling with fluorescent microspheres. We used antibody-mediated depletion protocols to dissect the relative contribution of T lymphocytes and monocytes to disease development.

**Results:**

In the hyperlipidemic environment the progression of TA was highly exacerbated and the inflammatory CD11b^+^CD115^+^Ly-6C^hi^ monocytes were preferentially recruited into the neointima. The number of macrophage-derived foam cells present in the grafts strongly correlated with plasma cholesterol and disease severity. Depletion of Ly-6C^hi^ monocytes and neutrophils significantly inhibited macrophage accumulation and disease progression. The accelerated monocyte recruitment occurs through a T cell-independent mechanism, as T cell depletion did not influence macrophage accumulation into the grafts.

**Conclusions:**

Our study identifies for the first time the involvement of inflammatory Ly-6C^hi^ monocytes into the pathogenesis of TA, particularly in conditions of hyperlipidemia. Targeted therapies modulating the recruitment and activation of these cells could potentially delay coronary allograft vasculopathy and improve long-term survival of heart transplant recipients.

## Highlights

► Inflammatory Ly-6C^hi^ monocytes infiltrate arterial allografts during TA development. ► Hyperlipidemia potently accelerates Ly-6C^hi^ monocyte recruitment into the neointima. ► TA severity correlates well with plasma cholesterol and macrophage accumulation. ► Macrophage-derived foam cell accumulation occurs independently of T lymphocytes. ► Ly-6C^hi^ monocyte and neutrophil depletion inhibits TA development.

## 1 Introduction

The development of cardiac allograft vasculopathy or transplant arteriosclerosis (TA) restricts long-term survival of heart transplant recipients [1,2]. TA is a fibroproliferative disease of the arterial vasculature, characterised by neointimal hyperplasia due to smooth muscle cell (SMC) proliferation and accumulation of extracellular matrix proteins. Unlike the focal atheromas characteristic of native atherosclerosis, TA lesions are concentric and diffusely spread throughout the coronary vasculature [3]. The most important trigger of TA development is the alloimmune-mediated injury of the graft vasculature, leading to endothelial activation and immune cell recruitment. Both innate and the adaptive immune mechanisms are involved in the pathogenesis of the disease [4].

Coronary allograft vasculopathy is the second most common cause of death in long-term survivors of heart transplantation, after malignancy [5]. Significant allograft vasculopathy develops in over 50% of heart allografts by 10 years after transplantation [5,6] and rapid initial disease progression is indicative of an increased risk for early myocardial infarction and mortality [7]. Unfortunately, due to the diffuse character of the disease, coronary revascularization procedures are inefficient and do not improve long-term survival [3].

Post-transplant hyperlipidemia accelerates the development of TA in cardiac allografts [8–10] and plasma cholesterol was found to be a strong independent predictor of major adverse cardiac events in heart transplant recipients [11]. A massive accumulation of lipid-rich lesions was observed in the coronary arteries of transplanted human hearts [12]. In animal studies hypercholesterolemia was associated with accelerated TA development [13–15]. However, the underlying pathogenic mechanisms linking hyperlipidemia with enhanced development of TA and the role of the different cellular populations in this process are largely unknown.

By using fully allogeneic arterial grafts transplanted into normolipidemic wild-type or hyperlipidemic ApoE^−/−^ mice we show that the hyperlipidemic recipients develop severe TA, characterized by intense foam cell infiltration into the neointima. Hyperlipidemia specifically enhances the recruitment of the inflammatory CD11b^+^CD115^+^Ly-6C^hi^ monocyte subset into the grafts and the macrophage accumulation strongly correlates with plasma cholesterol and the size of the lesions. Antibody-mediated depletion of Ly-6C^hi^ monocytes and neutrophils decreases intimal expansion and macrophage infiltration, suggesting an active role of these cells in the pathogenesis of the disease.

## 2 Materials and methods

Please consult the “Supplementary methods” section for a detailed description.

### 2.1 Animals

Sex- and age- matched male and female CBA.Ca (H2^k^), C57BL/6 (H2^b^), C57BL/6 ApoE^−/−^ (H2^b^) and C57BL/6 Rag^−/−^ (H2^b^) mice were used as vessel donors and/or transplant recipients as specified in the text.

### 2.2 Aorta transplantation and tissue analysis

Aortic segments harvested from CBA.Ca mice were transplanted into fully allogeneic C57BL/6 wild-type (wt) or ApoE^−/−^ recipients by an end-to-end infrarenal interposition technique as previously described [16]. The mice were fed either a regular mouse diet or a high fat diet (HFD) containing 21.4% fat and 0.15% supplementary cholesterol (Dietex International, Witham, UK). The HFD was initiated 2 weeks before transplantation and continued for the entire duration of the experiment. The grafts were harvested 21 days after transplantation unless otherwise specified. For morphometric analysis, 5 sections from each graft were collected at 200 μm intervals and stained with Miller's Elastin/van Gieson. The percentage of the lumen occupied by the neointima, termed intimal expansion, was calculated as % Intimal expansion = [*A*_I_/(*A*_I_+*A*_L_)] × 100, where *A*_I_ is the area of the neointima and *A*_L_ is the luminal area.

### 2.3 Immunostaining

Immunohistochemistry and immunofluorescence were performed on 10 μm frozen sections. Oxidized LDL (oxLDL) was detected as previously described [17] using a recombinant human IgG1 antibody binding to a specific ApoB-100 epitope (BioInvent AB, Lund, Sweden). T cells were stained with biotinylated rat anti-mouse CD4 and CD8 antibodies (eBioscience, Hatfield, UK) and macrophages with biotinylated rat anti-mouse CD68 (AbD Serotec, Oxford, UK) or CD11b (eBioscience, Hatfield, UK) antibodies as specified in the text. For immunofluorescence, macrophages were stained using biotinylated anti-mouse CD11b followed by a detection step with streptavidin-Alexa Fluor 594 (Invitrogen, USA). Smooth muscle cells were stained with Cy3-conjugated mouse anti-α actin (Sigma, USA).

### 2.4 *In vivo* monocyte labelling

The CD11b^+^CD115^+^Ly-6C^lo^ and the CD11b^+^CD115^+^Ly-6C^hi^ monocyte populations were differentially labelled *in vivo* using a protocol based on monocyte uptake of green fluorescent latex Fluoresbrite^®^YG microspheres (Polysciences Inc., USA) [18]. The monocytes were labelled 10 days after transplantation to allow for neointima development and the mice were sacrificed 4 days later.

### 2.5 Flow-cytometric analysis

Cellular preparations were stained using the following fluorochrome-conjugated antibodies: CD11b-PE, CD115-APC, CD3-PECy7, CD4-PB, CD8-PerCP and GR1-biotin followed by streptavidin-APCCy7 (eBioscience). The signal from the green fluorescent microspheres was detected in the FITC channel.

### 2.6 Statistics

All statistical analyses were performed using the non-parametric two-tailed Mann–Whitney test. Data are presented as mean ± SEM (standard error of the mean) or as box plots. The difference between the groups was considered to be statistically significant at *P* ≤ 0.05.

## 3 Experimental results

### 3.1 Hyperlipidemia accelerates TA

**Fig. 1:**
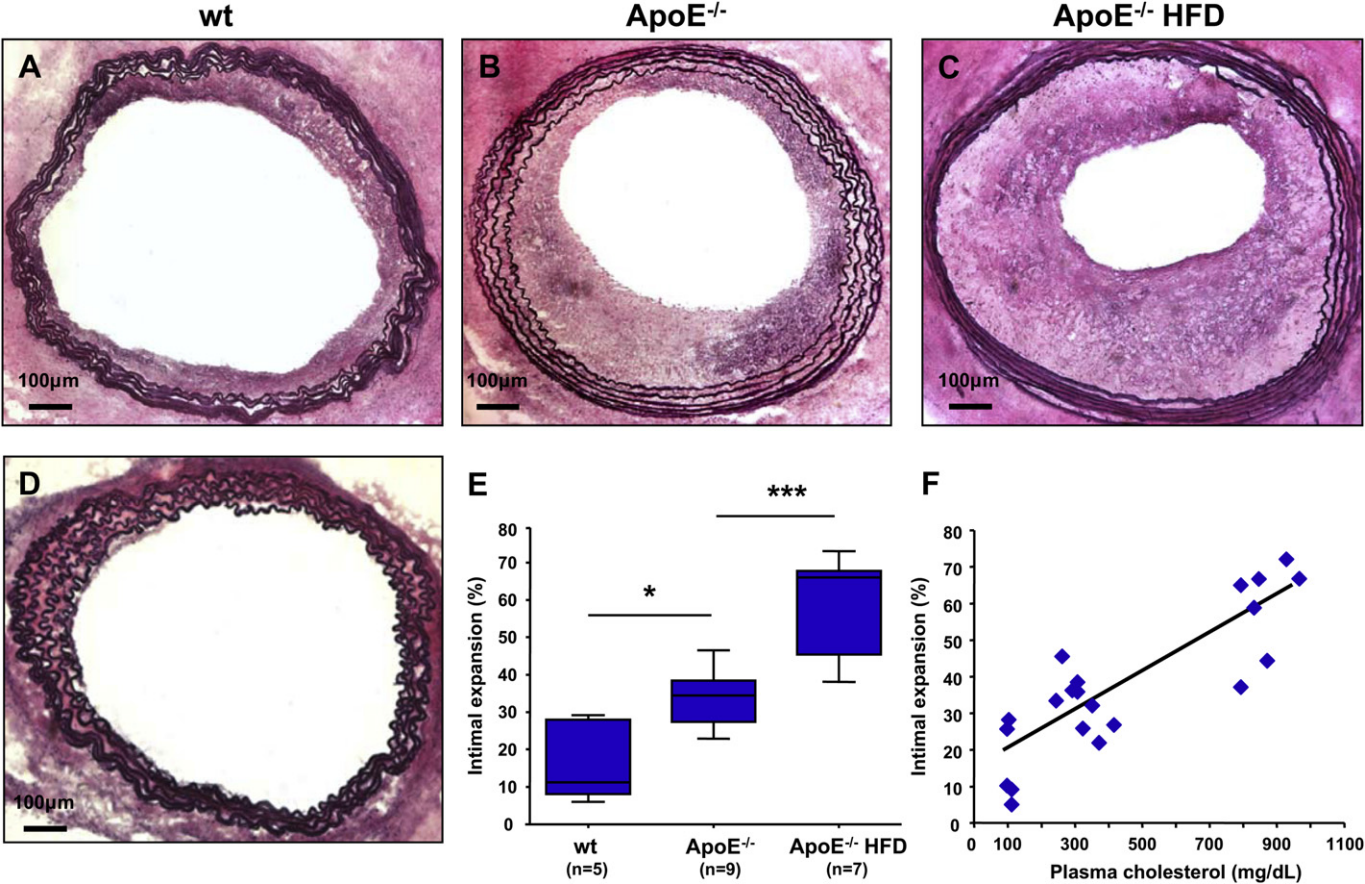
Hyperlipidemia accelerates the development of transplant arteriosclerosis (A–C). Representative photomicrographs of fully allogeneic CBA.Ca aortic grafts transplanted into C57BL/6 recipients: (A) wild-type recipient fed a regular mouse diet (wt); (B) ApoE^−/−^ recipient fed a regular mouse diet (ApoE^−/−^) and (C) ApoE^−/−^ recipient fed a high fat diet (ApoE^−/−^HFD). (D) ApoE^−/−^ graft transplanted into a syngeneic ApoE^−/−^ recipient on HFD. Elastin/van Giesson stain, the elastic laminae are purple and the cellular cytoplasm is pink. (E) TA severity in the wt (*n* = 5), ApoE^−/−^ (*n* = 9) and ApoE^−/−^HFD (*n* = 7) groups, expressed as intimal expansion. (F) Correlation between TA and total plasma cholesterol (*R*^2^ = 0.721, *P* < 0.0001). * *P* < 0.05, *** *P* < 0.001.

All of the allogeneic aortic grafts transplanted into immunocompetent mice developed TA, characterized by expansion of the neointima towards the vascular lumen (Fig. 1A–C). Disease progression was significantly accelerated in the ApoE^−/−^ recipients, both in mice fed regular mouse diet and those fed HFD, compared to the wt controls. Average TA increased by 100% in the ApoE^−/−^ group (33.9 ± 2.4%) and by 250% in the ApoE^−/−^HFD group (59.6 ± 4.9%) compared to the wt control (16.6 ± 4.7%) (Fig. 1E). TA did not develop in CBA.Ca aortas transplanted into immunodeficient C57BL/6 Rag^−/−^ mice (not shown) or in ApoE^−/−^ aortas transplanted into syngeneic ApoE^−/−^HFD recipients (Fig. 1D). These findings suggest that a competent anti-allogeneic adaptive immune response is required for the initiation and progression of TA and that the surgery-associated vascular trauma and hyperlipidemia alone do not initiate vascular lesions in the graft. The extent of intimal expansion strongly correlated with plasma levels of total cholesterol and LDL cholesterol (Fig. 1F and Supplemental table 1). We have also found a weaker correlation between intimal expansion and plasma triglyceride levels (Supplemental table 1). The total number of blood monocytes at the time of harvest was significantly elevated in the ApoE^−/−^ mice kept on HFD for a total of 5 weeks compared with the wt mice and the ApoE^−/−^ mice on a regular diet. The circulating neutrophil numbers did not differ significantly among the three groups (Supplemental table 2).

### 3.2 Hyperlipidemia triggers important histological changes of the neointima in the ApoE^−/−^ recipients

The ORO staining revealed a heavy lipid infiltration in aortic grafts harvested from the ApoE^−/−^HFD recipients (Supplemental Fig. 1B). OxLDL was absent in the neointima but was consistently found in the innermost layer of the media in the ApoE^−/−^HFD group, indicating the presence of a local pro-oxidative environment (Supplemental Fig. 1D). No lipid infiltration or oxLDL staining were detected in grafts harvested from wt recipients (Supplemental Figs. 1A and C).

In the ApoE^−/−^HFD mice the CD11b^+^ foam cells were the dominant infiltrating cell type in the grafts and their location coincided with the lipid deposits (Supplemental Fig. 1F). In the wt group CD11b^+^ cells lacked the foam cell appearance and were present throughout the lesion (Supplemental Fig. 1E). In the wt recipients neointima was almost exclusively formed by a thin layer of vascular SMC (Supplemental Fig. 1G). In contrast, in the ApoE^−/−^HFD mice the SMC formed a thick distinct layer located on the luminal side of the graft, which appears to be complementary to the foam cell layer (Supplemental Fig. 1F and H).

### 3.3 Macrophage accumulation into the grafts correlates with TA severity

**Fig. 2:**
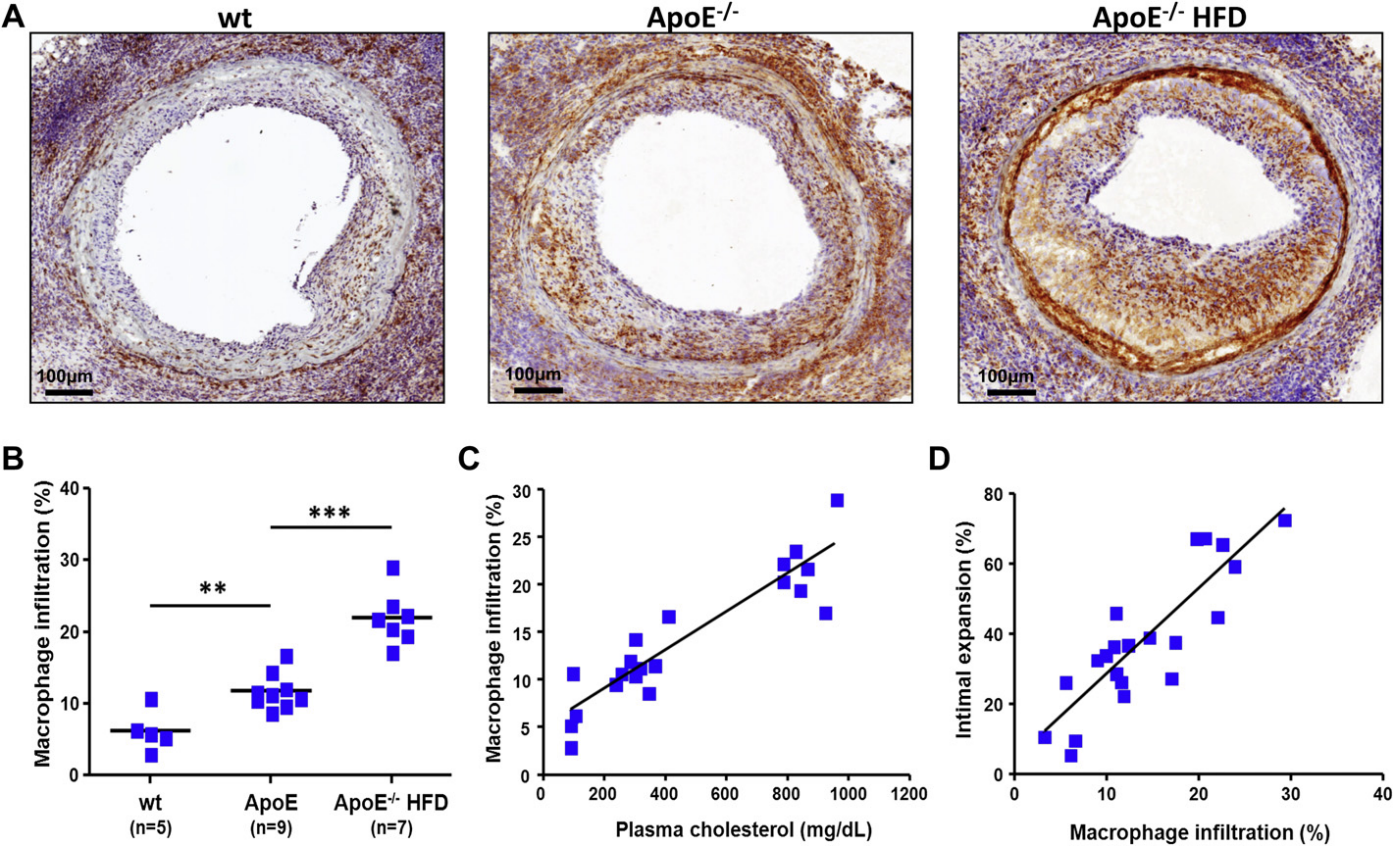
Macrophage accumulation into the neointima strongly correlates with plasma cholesterol and TA severity. (A) Representative micrographs of macrophage-specific CD68 staining of arterial allograft sections collected from wt, ApoE^−/−^ and ApoE^−/−^HFD recipients. (B) Macrophage accumulation in aortic allografts harvested from wt (*n* = 5), ApoE^−/−^ (*n* = 9) and ApoE^−/−^HFD (*n* = 7) hosts, expressed as percentage of the neointimal area. Macrophage recruitment is significantly increased in mice with high plasma cholesterol levels (C) (*R*^2^ = 0.847, *P* < 0.001) and correlates well with disease severity (D) (*R*^2^ = 0.751, *P* < 0.001). ** *P* < 0.01 *** *P* < 0.001.

The macrophage content of the lesions, measured as percentage CD68^+^ stained area reported to total lesion area, was doubled in the ApoE^−/−^ mice on normal mouse diet (11.8 ± 2.5%) and more than tripled in the ApoE^−/−^ mice on HFD (22.0 ± 3.8%) compared to the wt controls (6.3 ± 2.8%) (Fig. 2A and B). We found a highly significant direct correlation between total plasma cholesterol and the presence of macrophages in the neointima (*R*^2^ = 0.847, *P* < 0.001) (Fig. 2C). Furthermore, the intensity of macrophage infiltration strongly correlated with the extent of TA (*R*^2^ = 0.751, *P* < 0.001) (Fig. 2D).

### 3.4 T cell recruitment into the allograft is not dependent on hyperlipidemia and does not correlate with macrophage content

**Fig. 3:**
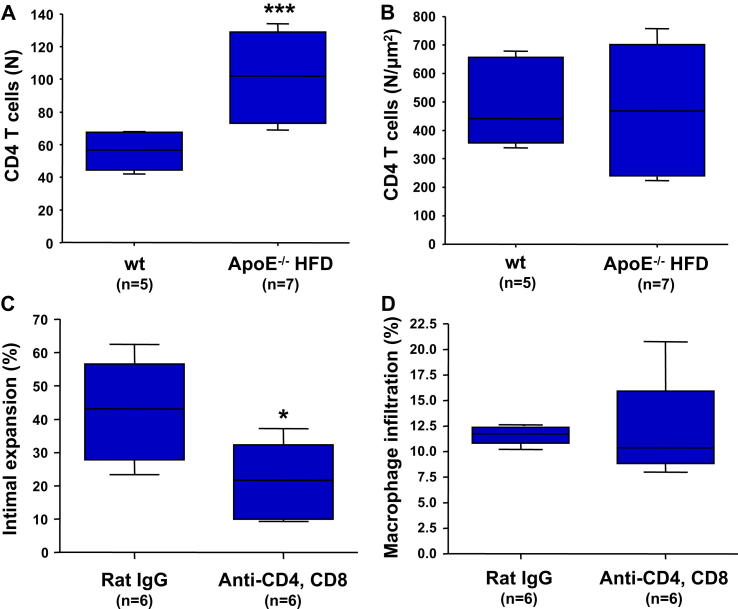
T cell depletion does not influence macrophage accumulation into the neointima. Quantification of CD4 T cell infiltration into allografts harvested from the normolipidemic wt (*n* = 5) and hyperlipidemic ApoE^−/−^HFD (*n* = 7) mice expressed as total number of infiltrating cells per section (A) and as number of cells reported to the area of the lesion (B). (C, D) Lesion development and macrophage recruitment in allografts harvested from ApoE^−/−^HFD recipients treated with depleting anti-CD4 and anti-CD8 antibodies (*n* = 6) compared to a rat IgG control group (*n* = 6). * *P* < 0.05, *** *P* < 0.001. HFD, high fat diet.

Both CD4^+^ and CD8^+^ T cells infiltrate the arterial grafts in all recipients (Supplemental Fig. 2). The Th1 subset of CD4^+^ T cells and their secreted cytokine IFNγ are major contributors to the development of vascular lesions in transplants [4]. In order to determine whether the accelerated lesion development in the ApoE^−/−^ mice is mediated through increased CD4^+^ T cell recruitment, we counted the numbers of CD4^+^ T cells infiltrating the neointima. The absolute number of CD4^+^ T cells was significantly increased in grafts harvested from the ApoE^−/−^HFD mice (Fig. 3A). However, there was no difference between the groups when we reported the number of CD4^+^ T cells relative to the size of the lesion, indicating a similar recruitment rate of these cells into the grafts in the normolipidemic and the hyperlipidemic environments (Fig. 3B).

In order to test whether the increased monocyte recruitment is T cell dependent, we treated a group of ApoE^−/−^HFD recipients with a combination of depleting anti-CD4 (YTA 3.1, 100 μg) and anti-CD8 (YTS 169, 200 μg) antibodies administered i.v. More than 95% depletion of T cells was achieved within 1 week after injection (Supplemental Figs. 3A and B). T cell depletion did not significantly influence the total numbers of circulating monocytes and neutrophils in mouse blood (Supplemental Figs. 3C and D). The antibodies were administered weekly starting at 7 days before transplantation. ApoE^−/−^HFD mice receiving 300 μg non-specific rat IgG served as the control group. T cell depletion induced a 50% decrease in lesion size compared to the controls (*P* < 0.05) (Fig. 3C). The TA reduction was surprisingly modest considering that the recipient peripheral T cell pool was maintained at below 5% of the original size. These data indicate that while a small number of T cells are sufficient to trigger the development of TA, additional factors play important roles in the pathogenic process. We found an equal accumulation of macrophages inside the lesions in the 2 groups (Fig. 3D), suggesting that the increased macrophage recruitment in the hyperlipidemic recipients is preferentially governed by T cell-independent mechanisms.

### 3.5 Hyperlipidemia accelerates the recruitment of inflammatory Ly-6C^hi^ monocytes into the graft

Monocytes can be functionally divided in 2 main subpopulations: inflammatory CD11b^+^CD115^+^Ly-6C^hi^ (GR1^hi^) and resident CD11b^+^CD115^+^Ly-6C^lo^ (GR1^lo^) [19]. By using an *in vivo* protocol based on monocyte uptake of fluorescent latex microspheres [18] we differentially labelled the two populations in 2 groups of wt and 2 groups of ApoE^−/−^HFD recipients of CBA.Ca aortic grafts (Supplemental Fig. 4). The monocytes were labelled 10 days after transplantation and the arterial grafts were harvested 4 days later as the 2 monocyte populations remain distinctly labelled within this time frame [18].

**Fig. 4:**
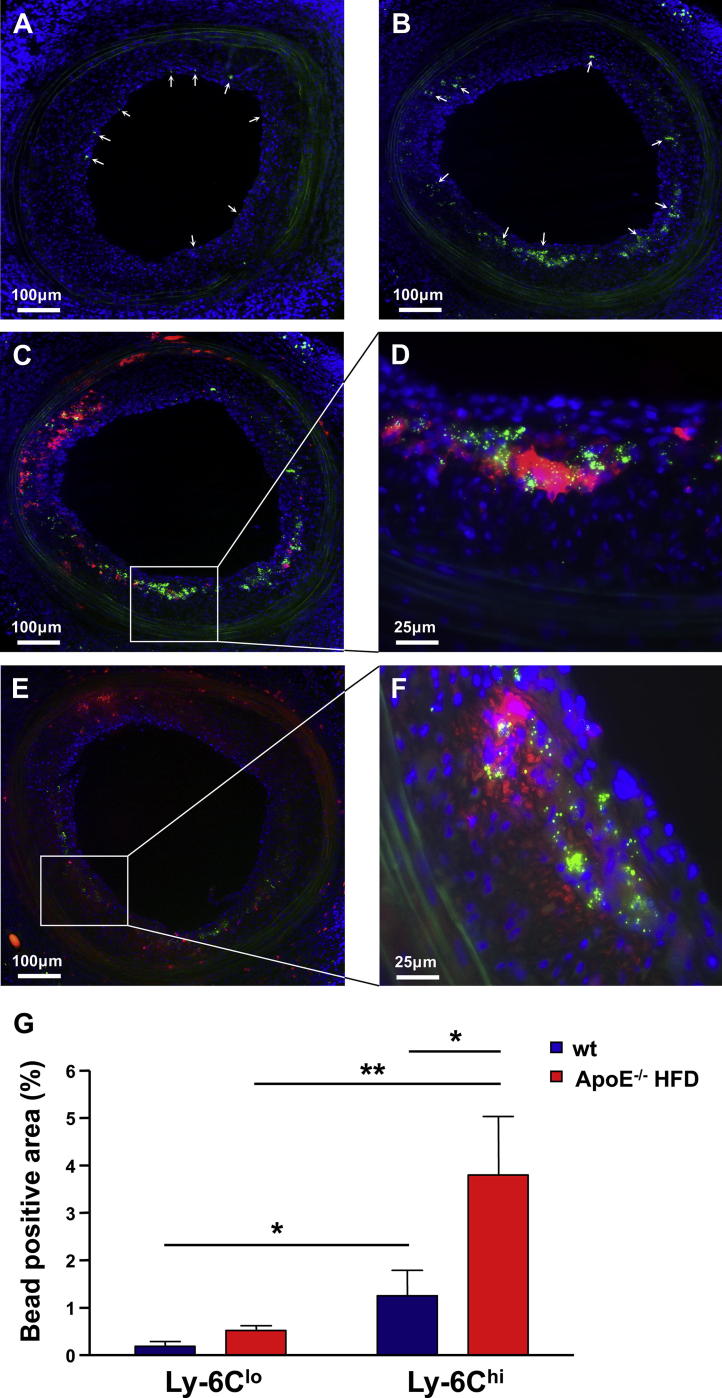
Hyperlipidemia accelerates the recruitment of inflammatory Ly-6C^hi^ monocytes into the arterial grafts. The fluorescent photomicrographs in (A) and (B) show the presence of green fluorescent microspheres in arterial allografts harvested from ApoE^−/−^HFD mice in which the Ly-6C^lo^ (GR1^lo^) (A) and the Ly-6C^hi^ (GR1^hi^) (B) monocytes were labelled. The latex microspheres were injected 10 days after transplantation and the grafts were harvested 4 days later. Blue is DAPI nuclear stain, the elastic laminae in the media are green autofluorescent and the white arrows indicate the location of the fluorescent microspheres. (C, D) Red immunoflourescent staining of CD11b^+^ (C) and GR1^+^ (D) cells in graft sections from the ApoE^−/−^HFD Ly-6C^hi^ group showing infiltration of both microsphere^+^ and microsphere^−^ CD11b^+^ and GR1^+^ cells into the neointima, with the majority of the latex microspheres located inside the cells. (D, F) Four times enlargement of the marked areas in (C) and (E) demonstrating the intracellular location of the microspheres. (G) Fluorescent microsphere infiltration into the neointima of allogeneic aortic grafts harvested from 2 groups of wt recipients fed a regular mouse diet and 2 groups of ApoE^−/−^HFD recipients in which the Ly-6C^hi^ and the Ly-6C^lo^ monocytes were differentially labelled (*n* = 6 mice per group). Microsphere density was quantified as percentage microsphere^+^ area per total lesion area. * *P* < 0.05, ** *P* < 0.01. HFD, high fat diet.

The fluorescent microspheres were present in the arterial grafts in all 4 groups of mice at the time of harvest (not shown). Whereas the Ly-6C^lo^ monocytes were preferentially located under the endothelium, the inflammatory Ly-6C^hi^ monocytes penetrated deep into the neointima through to the internal elastic lamina (Fig. 4A and B). Fluorescent anti-CD11b and anti-GR1 counterstaining of the grafts revealed that the microspheres are mainly located inside the infiltrating CD11b^+^ and GR1^+^ cells and that both microsphere^+^ and microsphere^−^ CD11b^+^ and GR1^+^ cells are present into the neointima (Fig. 4C–F). We quantified the microsphere content of the neointima as green fluorescent positive area per total lesion area. Significantly more fluorescent microspheres infiltrated the grafts in recipients where the Ly-6C^hi^ monocytes were labelled compared to their Ly-6C^lo^ counterparts both in wt (mean ± SEM 0.55 ± 0.29% vs. 0.09 ± 0.03%, *P* < 0.05) and ApoE^−/−^HFD mice (3.23 ± 0.88% vs. 0.29 ± 0.04%, *P* < 0.01) (Fig. 4G). The hyperlipidemic environment led to accelerated recruitment of Ly-6C^hi^ monocytes into the grafts harvested from the ApoE^−/−^HFD mice compared to wt controls (*P* < 0.05), whereas there was no significant difference regarding Ly-6C^lo^ monocyte infiltration between the two groups (Fig. 4G).

### 3.6 Depletion of GR1^+^ inflammatory monocytes and neutrophils decreases TA and intra-graft macrophage accumulation

**Fig. 5:**
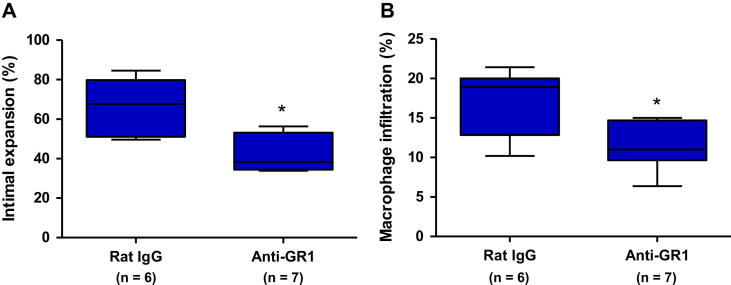
Depleting anti-GR1 antibody treatment decreases TA development and macrophage accumulation into the neointima (A) Lesion development in CBA.Ca grafts harvested from ApoE^−/−^HFD recipients treated with depleting anti-GR1 antibodies (*n* = 7) or rat IgG (*n* = 6). (B) Macrophage content in the neointima of grafts harvested from the anti-GR1 and the control IgG groups. * *P* < 0.05.

In order to determine whether the inflammatory monocytes play a pathogenic role in disease progression we treated a group of ApoE^−/−^HFD mice receiving CBA.Ca arterial grafts with depleting rat anti-mouse anti-GR1 antibodies (clone RB6-8C5; 200 μg/injection) administered i.p. every second day starting at one day before transplantation. The RB6-8C5 antibody binds both Ly-6C on monocytes as well as Ly-6G on neutrophils. Our preliminary experiments showed that the antibody depletes neutrophils and the Ly-6C^hi^ (GR1^hi^) monocyte population, confirming previously published data by Daley et al. [20] (Supplemental Fig. 5). The anti-GR1 antibody treatment significantly decreased intimal expansion and the macrophage content of the lesions by approximately 35% as compared with a rat IgG control group (Fig. 5).

## 4 Discussion

By using a mouse model of arterial transplantation we demonstrate for the first time that the inflammatory Ly-6C^hi^ monocytes are involved in the pathogenesis of TA and that hyperlipidemia potently accelerates the recruitment of this distinct cellular population into the grafts. Antibody-mediated depletion of Ly-6C^hi^ monocytes and neutrophils decreases macrophage accumulation and lesion development, suggesting an active involvement of these cells in the pathogenesis of the disease. The macrophage-derived foam cells are a major component of the neointima in hyperlipidemic hosts. We found significant correlations between plasma cholesterol, macrophage accumulation and lesion size, suggesting that the increased Ly-6C^hi^ monocyte recruitment may act as a link between hypercholesterolemia and the accelerated lesion development. Interestingly, this mechanism seems to be independent of T cell-mediated alloimmunity, as the density of T cells infiltrating the graft remained unchanged in the hyperlipidemic versus the normolipidemic hosts and T cell depletion did not influence macrophage accumulation.

The accelerated TA development in cardiac and vessel allografts in hyperlipidemic animals was previously shown to be associated with increased local expression of VCAM-1 and ICAM-1, enhanced neovascularization and higher levels of TNFα in SMCs [13–15]. Although these studies acknowledged the presence of infiltrating lipids and foam cells, they suggested that the accelerated disease progression is mediated by increased SMC accumulation into the neointima [14,15]. Our results do not contradict this hypothesis, as we have also found a thicker neointimal SMC layer in the arterial grafts from the ApoE^−/−^HFD group compared to wt controls. However, the strong direct correlation between cholesterol levels, macrophage infiltration and lesion size suggests that monocytes/macrophages are a major contributor to the enhanced lesion development in the hyperlipidemic environment. In grafts harvested from the ApoE^−/−^HFD recipients, the CD68^+^ immunostained area accounts for up to 30% of the total size of the lesion but, as the detection antibody only binds to cellular membrane, the total area occupied by lipid-loaded foam cells is in fact much larger. In contrast, macrophages occupy only 6% of the neointima in the lesions developed in normolipidemic hosts.

Similar to native atherosclerosis, lipid infiltration and oxLDL accumulation inside the vascular wall seems to trigger the recruitment of monocytes in an attempt to clear the infiltrating lipids from the tissue [21]. We found that the presence of oxLDL is exclusively confined to the inner layers of the media. This is a distinctive feature of TA, as in native atherosclerosis the oxLDL-associated epitopes can be detected throughout the atheroma [17]. OxLDL is pro-inflammatory and immunogenic and has been shown to trigger accumulation and activation of macrophages, T cells and SMC [21–23]. It appears that the accelerated foam cell accumulation and SMC proliferation occur in parallel through local mechanisms triggered by the increased lipid infiltration and oxidation.

It has previously been suggested that macrophages might be involved in the pathogenesis of TA, as phagocyte depletion inhibited development of cardiac allograft vasculopathy in a mouse model of heart transplantation [24]. We demonstrate that the Ly-6C^hi^ monocytes are preferentially recruited into the neointima compared to their Ly-6C^lo^ counterparts both in the normolipidemic and in the hyperlipidemic hosts and that hyperlipidemia specifically accelerates this process. Our data extend previous studies by other groups showing that Ly-6C^hi^ monocytes preferentially develop into plaque macrophages and dendritic cells in atherosclerotic lesions [18,25] and are the main monocyte population associated with rejection of murine heart allografts [26]. Antibody-mediated depletion of Ly-6C^hi^ monocytes and neutrophils significantly decreased macrophage accumulation and delayed disease progression. It is difficult to speculate whether these effects are mainly due to Ly-6C^hi^ monocyte depletion or whether neutrophils also play a role. The involvement of neutrophils in the pathogenesis of TA is currently unknown and certainly warrants further investigation. In the case of native atherosclerosis, recruitment of inflammatory Ly-6C^hi^ monocytes into the atherosclerotic plaques requires particular chemokine–chemokine receptor interactions, involving CXCR1 in addition to CCR2 and CCR5 [18]. It remains to be determined whether similar pathways are involved in the recruitment of inflammatory monocytes into the arterial grafts. Identifying the key chemokines and chemokine receptors involved in this process might provide treatment targets for specifically restricting the involvement of Ly-6C^hi^ monocytes in the development of allograft vasculopathy.

T cells play a major role in the pathogenesis of TA. Interestingly, the density of CD4^+^ T cells inside the lesions was similar in grafts harvested from ApoE^−/−^HFD mice and wt controls. Treatment of ApoE^−/−^HFD recipients with depleting anti T cell antibodies led to significantly smaller lesions compared to controls, but did not influence the rate of macrophage accumulation. These observations suggest that the effect of hyperlipidemia on lesion formation is not T cell mediated and that the accumulating macrophages do not accelerate disease by triggering an increased recruitment of T cells into the graft. Our results are in line with previous findings demonstrating that inhibition of macrophage phagocytosis and antigen-presentation with gadolinium chloride did not delay the development of coronary allograft vasculopathy in mice [24].

The recruitment of Ly-6C^hi^ monocytes into the neointima in the ApoE^−/−^ mice on a HFD occurs at a much faster pace than that observed in native atherosclerosis [18], confirming that the pathogenesis of TA employs an accelerated inflammatory and hyperplastic process compared to native atheromas [3]. These findings correlate with data from previous experimental and clinical studies that have demonstrated a much higher accumulation of lipids over a shorter time period in the coronary arteries of transplanted hearts compared to native atherosclerosis [12,13]. A potential drawback of our model is that plasma lipid levels are much higher in the ApoE^−/−^ mice fed a HFD compared to human subjects, and therefore we cannot rule out that this might have exacerbated effects on lesion progression. Additionally, our data and studies by other groups demonstrate a marked escalation in the total numbers of circulating monocytes in the ApoE^−/−^ mice fed a HFD, mainly due to an increase in the Ly-6C^hi^ population [18,25–27]. Consecutively, it is possible that the increased availability of circulating inflammatory monocytes might have contributed to the highly exacerbated disease progression in the ApoE^−/−^HFD group. However, the correlation between plasma cholesterol levels, macrophage accumulation and the size of the neointima was also valid in the ApoE^−/−^ mice fed a regular mouse diet, which have much lower lipid levels and similar total and Ly-6C^hi^ monocyte counts as the wt controls [25]. These observations suggest that the local environment of the neointima, characterised by intense lipid infiltration and oxidative modifications, plays an important role in the preferential recruitment of the inflammatory monocytes into the arterial allografts. The histological similarity between the grafts harvested from the ApoE^−/−^ recipients and the appearance of coronary arteries in necropsy specimens from transplanted human hearts [12] indicates that this is an accelerated model which might accurately reflect the events that occur at a slower rate in the clinical setting. We propose the ApoE^−/−^ mice as a more appropriate recipient strain for studies of transplant-associated vascular pathology than wt mice, as the wt models do not reflect the hyperlipidemic conditions that often occur in transplant patients.

## 5 Conclusions

To our knowledge, this is the first report describing the important role played by the inflammatory Ly-6C^hi^ monocytes in the pathogenesis of TA. Our data provide a possible mechanistic explanation for the previously reported correlation between hyperlipidemia, severe coronary vasculopathy and increased prevalence of adverse cardiac events in heart transplant recipients [8–11]. The accumulation of macrophage-derived foam cells into the arterial allografts occurs independently of T cells and correlates well with plasma cholesterol levels and the size of the lesions. From a clinical perspective, our findings support current guidelines recommending early post-transplant lipid-lowering therapy. Statins have been shown to prevent hypercholesterolemia-associated monocytosis in mice [25] and to delay coronary allograft vasculopathy in humans, significantly improving survival rates of cardiac transplant recipients [28]. While the current immunosuppressive therapies are mainly directed against lymphocyte activation and proliferation, other cellular populations have largely been overlooked so far. The ability of the immunosuppressive drug rapamycin to inhibit monocyte infiltration into the arterial wall [29] might partly explain its demonstrated superior efficiency compared to other immunosupressants for the prevention of coronary allograft vasculopathy [30]. Our study identifies the Ly-6C^hi^ inflammatory monocytes as new potential therapeutic targets for further improvement of long-term survival in heart transplant recipients.

## Sources of financial support

The study was supported by grants from the Garfield Weston Trust, The Wellcome Trust and British Heart Foundation. Dr. Schiopu was supported by the Swedish Heart and Lung Foundation and the Swedish Research Council. Dr. Nadig was an International Society for Heart and Lung Transplantation Research Fellow. The funding source(s) were not directly involved in study design, collection, analysis and interpretation of data, writing of the report or in the decision to submit the article for publication.

## Conflicts of interest and relationships with the industry

None.
