# Supplementary material for: Inflammatory Ly-6Chi monocytes play an important role in the development of severe transplant arteriosclerosis in hyperlipidemic recipients
Source: Atherosclerosis. 2012 Aug;223(2):291–8. doi: 10.1016/j.atherosclerosis.2012.05.010 (PMC3423631; doi:10.1016/j.atherosclerosis.2012.05.010)
Supplement: Supplementary file 1 [file mmc1.pdf]

SUPPLEMENTAL MATERIAL

Detailed methods

Animals

Sex- and age-matched male and female CBA.Ca ($H2^k$), C57BL/6 ($H2^b$), C57BL/6 $ApoE^{-/-}$ ($H2^b$), and C57BL/6 $Rag^{-/-}$ ($H2^b$) mice were used as vessel donors and/or transplant recipients as specified in the text. The $ApoE^{-/-}$ mice were purchased from The Jackson Laboratory, UK (stock number: 002052) and were backcrossed for at least 10 generations. The animals were bred and maintained in the Biomedical Services Unit at the John Radcliffe Hospital (Oxford, U.K.) and were treated in strict accordance with the Home Office Animals (Scientific Procedures) Act of 1986 and the directive 2010/63/EU of the European Parliament. All the experimental protocols were approved by the Committee on Animal Care and Ethical Review at Oxford University.

Aorta transplantation and tissue analysis

Aortic segments harvested from CBA.Ca mice were transplanted into fully allogeneic C57BL/6 wild-type (wt) or $ApoE^{-/-}$ recipients by an end-to-end infrarenal interposition technique as previously described.[1] Previous studies in our laboratory have shown that this model accurately reflects the vascular changes that occur in transplanted solid organs in animal studies.[1] Previous to surgery the animals were anesthetised using medetomidine/ketamine (1mg/kg and 80mg/kg, respectively) and buprenorphine (0.6mg/kg) injected s.c. The donor mice were anti-coagulated with 1mL heparinized saline (400U/mL) injected into the inferior vena cava and an additional

1mL injected into the left ventricle of the heart. The segment of the thoracic aorta between the left subclavian artery and the diaphragmatic hiatus was dissected free of connective tissue and fat and stored in ice-cold heparinized saline. In the recipient mice access to the abdominal aorta was obtained through a midline incision and viscera were retracted from the abdominal cavity and kept moist throughout the surgery. We separated the aorta from the inferior vena cava and 2 microvascular clamps were placed on the aorta immediately below the renal arteries and above the iliac bifurcation, respectively. The vessel was then transected between the clamps and a segment of the donor aorta of appropriate size was fitted within the gap. The origins of the spinal arteries emerging from the graft were closed using a low-power electrical cautery. We attached the graft to the recipient aorta by end-to-end anastomoses using a single, interrupted technique with 10-0 monofilament nylon suture (6-8 sutures for each anastomosis). Following anastomosis we removed the distal clamp first followed by the proximal clamp and achieved hemostasis by light lateral pressure on the anastomosis with sterile cotton swabs. The intestines were then repositioned and 1 ml of normal saline was instilled into the peritoneal cavity. The abdominal musculature and the skin were closed in 2 layers by continuous sutures. Following surgery we injected subcutaneously 0.3 mL atipamezole hydrochloride (100µg/mL) for reverse anesthesia and 1 mL warm saline to maintain fluid balance. The mice were placed inside a warm cabinet until they awoke.

The mice were fed either a regular mouse diet or a high fat diet (HFD) containing 21.4% fat and 0.15% supplementary cholesterol (Dietex International, Witham, UK). The HFD was initiated 2 weeks before transplantation and continued for the entire duration of the experiment. The mice were sacrificed by exsanguination under anaesthesia and the grafts were harvested 21 days after

transplantation unless otherwise specified, snap-frozen in OCT (Sakura Finetek, The Netherlands) and cryostat sectioned at a 10µm thickness. For morphometric analysis, 5 sections from each graft were collected at 200µm intervals, fixed in cold acetone and paraformaldehyde and stained with Miller's Elastin/van Gieson for 8 minutes and 1 minute, respectively. The percentage of the lumen occupied by the neointima, termed intimal expansion, was calculated using the following formula: % Intimal expansion = (A_I/A_I+A_L) x 100, where A_I is the area of the neointima and A_L is the luminal area. For lipid assessment the sections were fixed in Histochoice (Amresco, USA) and stained with 0.3% Oil Red O (ORO) solution in isopropanol for 7 minutes.

Immunostaining

Immunohistochemistry and immunofluorescence were performed on sections fixed in cold acetone and blocked with 10% mouse serum for 30 minutes in room temperature. Oxidized LDL (oxLDL) was detected as previously described[2] using a recombinant human IgG1 antibody binding to a specific ApoB-100 epitope as primary antibody (BioInvent AB, Lund, Sweden) and a biotinylated mouse anti-human IgG1 secondary antibody (BD Biosciences, USA). T cells were stained with biotinylated rat anti-mouse CD4 and CD8 antibodies (eBioscience, Hatfield, UK) and macrophages with biotinylated rat anti-mouse CD68 (AbD Serotec, Oxford, UK) or CD11b (eBioscience, Hatfield, UK) antibodies as specified in the text. Colour development was performed using an ABC immunoperoxidase kit (Vector Laboratories, USA) and diaminobenzydine (Sigma, USA). For immunofluorescence, macrophages were stained using biotinylated anti-mouse CD11b followed by a detection step with streptavidin-Alexa Fluor 594 (Invitrogen, USA). Smooth muscle cells were stained with Cy3-conjugated mouse anti-α actin

(Sigma, USA). Macrophage accumulation was quantified as percentage macrophage stained area per total lesion area. The number of infiltrating CD4 T cells was counted manually and reported to lesion area. The results represent the average values of five sections per mouse, collected at 200µm intervals. Image analysis was performed using the Image ProPlus software (MediaCybernetics, USA).

***In vivo* monocyte labelling**

The patrolling $CD11b^+CD115^+Ly\text{-}6C^{lo}$ and the inflammatory $CD11b^+CD115^+Ly\text{-}6C^{hi}$ monocyte populations were differentially labelled *in vivo* using a protocol based on monocyte uptake of green fluorescent latex Fluoresbrite® YG 0.50 µm microspheres (Polysciences Inc., USA).[3] Briefly, 200µL of 1/25 diluted microspheres were injected 10 days after transplantation into the tail vein of wt recipients fed a regular mouse diet or $ApoE^{-/-}$ mice fed a HFD. This protocol leads to almost exclusive labelling of the $Ly\text{-}6C^{lo}$ monocyte population between days 2-4 after injection, as assessed by flow-cytometry.[3] Temporary monocyte depletion using 200µL of clodronate-loaded liposomes injected i.v. 18 hours prior to microsphere administration shifts the distribution of microspheres towards the $Ly\text{-}6C^{hi}$ population during the same time frame. Cl2MDP (or clodronate) was a gift of Roche Diagnostics GmbH, Mannheim, Germany. All mice in this experiment were sacrificed 4 days after microsphere injection and aortic grafts, blood and spleen were harvested for analysis. The aortic sections were fixed in Histochoice prior to staining. Acetone or formaldehyde fixation was avoided in order to preserve the intracellular location of the microspheres.

Flow-cytometric analysis

Blood and spleen were collected from the recipient mice on the day of sacrifice. Blood was collected in heparin-coated tubes and kept on ice. Following centrifugation at 1500 rpm on a tabletop centrifuge plasma was frozen at -80°C until analysis. Single cell suspension in PBS buffer containing 2% heat inactivated fetal calf serum were prepared from the spleens using 70 μm cell-strainers (BD Biosciences). Red blood cells were lysed with Trizma base ammonium chloride (TBAC) solution for 5 minutes at room temperature. Cellular preparations were stained for flow-cytometric analysis using the following fluorochrome-conjugated antibodies: CD11b-PE, CD115-APC, CD3-PECy7, CD4-PB, CD8-PerCP and GR1 (Ly6C)-biotin followed by streptavidin-APCCy7. All antibodies were purchased from eBioscience. The signal from the green fluorescent microspheres was detected in the FITC channel. The analyses were performed on a FACSAria instrument (BD Biosciences) and the data was analysed using the FACSDiva software (BD Biosciences).

Plasma lipids measurement

Plasma total cholesterol, HDL cholesterol and triglycerides were measured by an automated technique at the Clinical Biochemistry department of the John Radcliffe Hospital in Oxford. The values for LDL cholesterol were calculated using the Friedwald formula: LDL cholesterol = Total cholesterol – HDL cholesterol – (Triglycerides/5).

Statistics

All statistical analyses were performed using the non-parametric two-tailed Mann-Whitney test. Data are presented as mean ± SEM (standard error of the mean) or as box plots showing median

and 25th and 75th percentiles as well as the highest and lowest values. The difference between the groups was considered to be statistically significant at $p \leq 0.05$.

Supplemental references

[1] Ensminger, SM, Billing, JS, Morris, PJ, et al., Development of a combined cardiac and aortic transplant model to investigate the development of transplant arteriosclerosis in the mouse, J Heart Lung Transplant, 2000;19:1039-1046.

[2] Schiopu, A, Bengtsson, J, Soderberg, I, et al., Recombinant human antibodies against aldehyde-modified apolipoprotein B-100 peptide sequences inhibit atherosclerosis, Circulation, 2004;110:2047-2052.

[3] Tacke, F, Alvarez, D, Kaplan, TJ, et al., Monocyte subsets differentially employ CCR2, CCR5, and CX3CR1 to accumulate within atherosclerotic plaques, J Clin Invest, 2007;117:185-194.

Supplemental table 1. Correlation between plasma lipid levels and TA

Mouse group	Number of mice	Neointimal expansion (%)	Total cholesterol (mg/dL)	LDL cholesterol (mg/dL)	Triglycerides (mg/dL)
**wt**	5	16.6 ± 4.7	93 ± 3	38 ± 4	65 ± 13
**ApoE$^{-/-}$**	9	33.9 ± 2.4	308 ± 18	280 ± 17	53 ± 10
**ApoE$^{-/-}$HFD**	7	59.6 ± 4.9	850 ± 25	784 ± 26	188 ± 24
**Correlation coefficient# (r^2)**			0.721***	0.725***	0.419*

All values are presented as mean ± SEM; wt, wild-type; HFD, high fat diet; # Describes the correlation between the extent of intimal expansion and plasma concentration of the respective lipid fraction at the time of harvest.

* *P*<0.05, *** *P*<0.001

Supplemental table 2. Circulating monocyte and neutrophil numbers

**Mouse group**	**Number of mice**	**Total monocytes in blood (millions/mL)**	**Total neutrophils in blood (millions/mL)**
**wt**	5	0.17 ± 0.06	0.43 ± 0.09
**ApoE$^{-/-}$**	9	0.21 ± 0.05	0.43 ± 0.13
**ApoE$^{-/-}$HFD**	7	0.46 ± 0.07***	0.48 ± 0.18

All values are presented as median ± SD; wt, wild-type; HFD, high fat diet; *** *P*<0.001 vs wt and ApoE$^{-/-}$

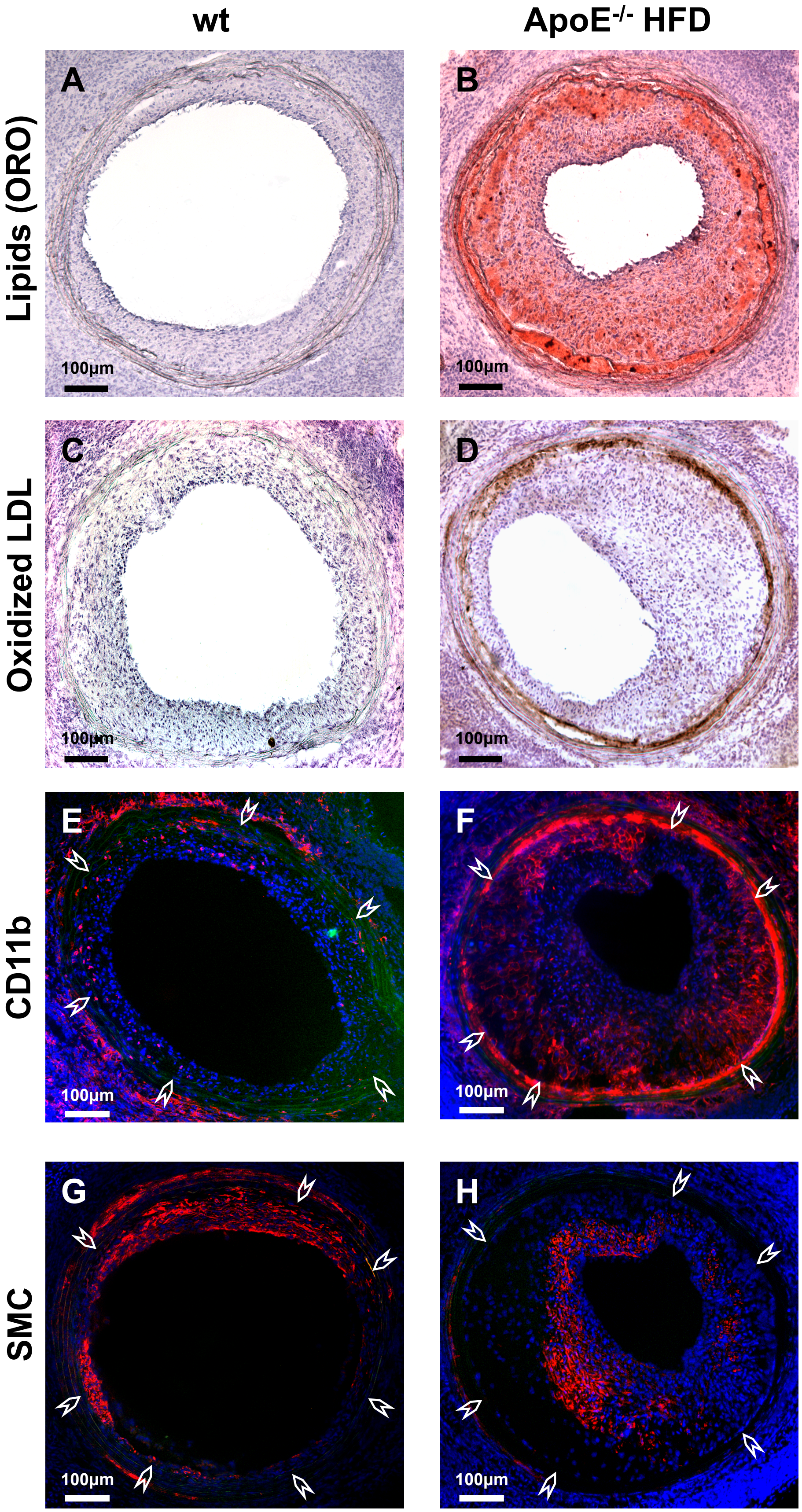

wt
ApoE-/- HFD
Lipids (ORO)
Oxidized LDL
CD11b
SMC
A
B
C
D
E
F
G
H
100µm

Supplemental figure 1. Comparative histological analysis of arterial grafts harvested from normolipidemic and hyperlipidemic hosts

(A-B) ORO lipid staining of allogeneic CBA.Ca aortic grafts harvested from a wt and an $ApoE^{-/-}$ HFD recipient. (C-D) Immunohistochemical staining of oxLDL epitopes (brown). (E-H) Red immunofluorescent staining demonstrating the location of macrophages (CD11b staining; E-F) and smooth muscle cells (alpha-actin staining; G-H). The photomicrographs are representative of $n \geq 5$ mice per group. The white arrows indicate the location of the internal elastic lamina. ORO, Oil Red O; wt, wild-type; HFD, high fat diet; oxLDL, oxidized LDL.

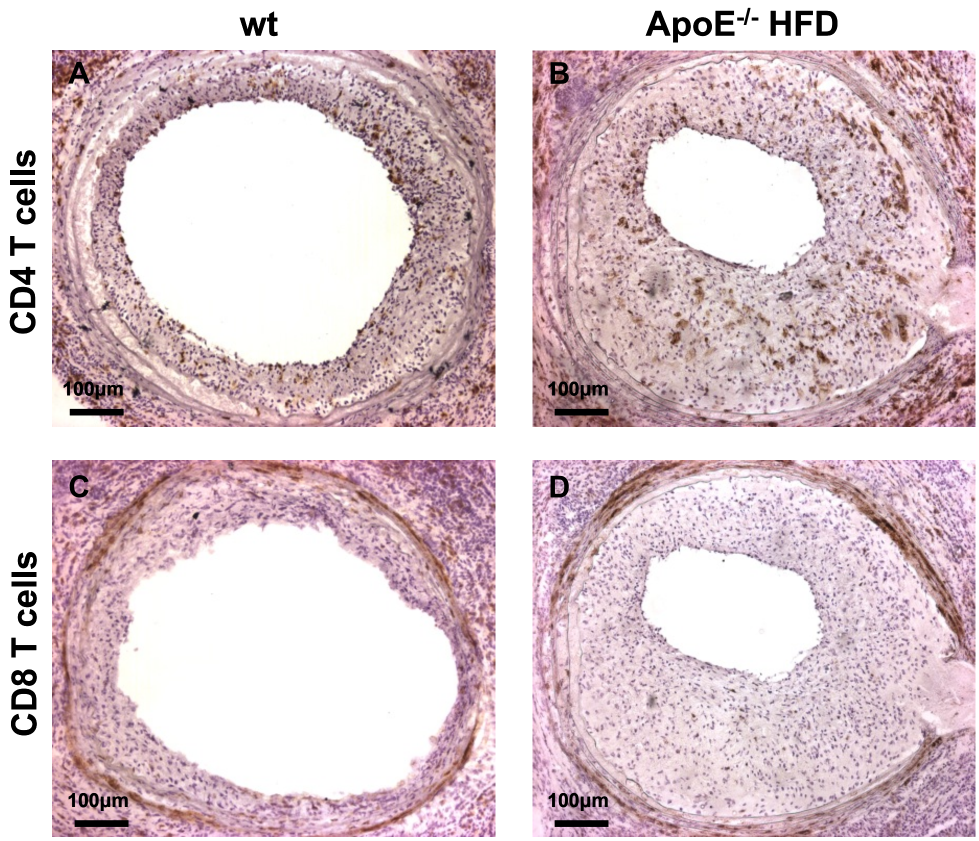

Supplemental figure 2. CD4 and CD8 T cells infiltrate the allografts

Representative photomicrographs demonstrating the presence of CD4 (A, B) and CD8 (C, D) T cells in allografts harvested 21 days after transplantation from a normolipidemic wt mouse fed a regular diet (A, C) and a hyperlipidemic ApoE$^{-/-}$ mouse fed a HFD (B, D). The CD4 and CD8 T cells stain brown with DAB and the sections were counterstained with haematoxylin. HFD, high fat diet; DAB, diaminobenzidine.

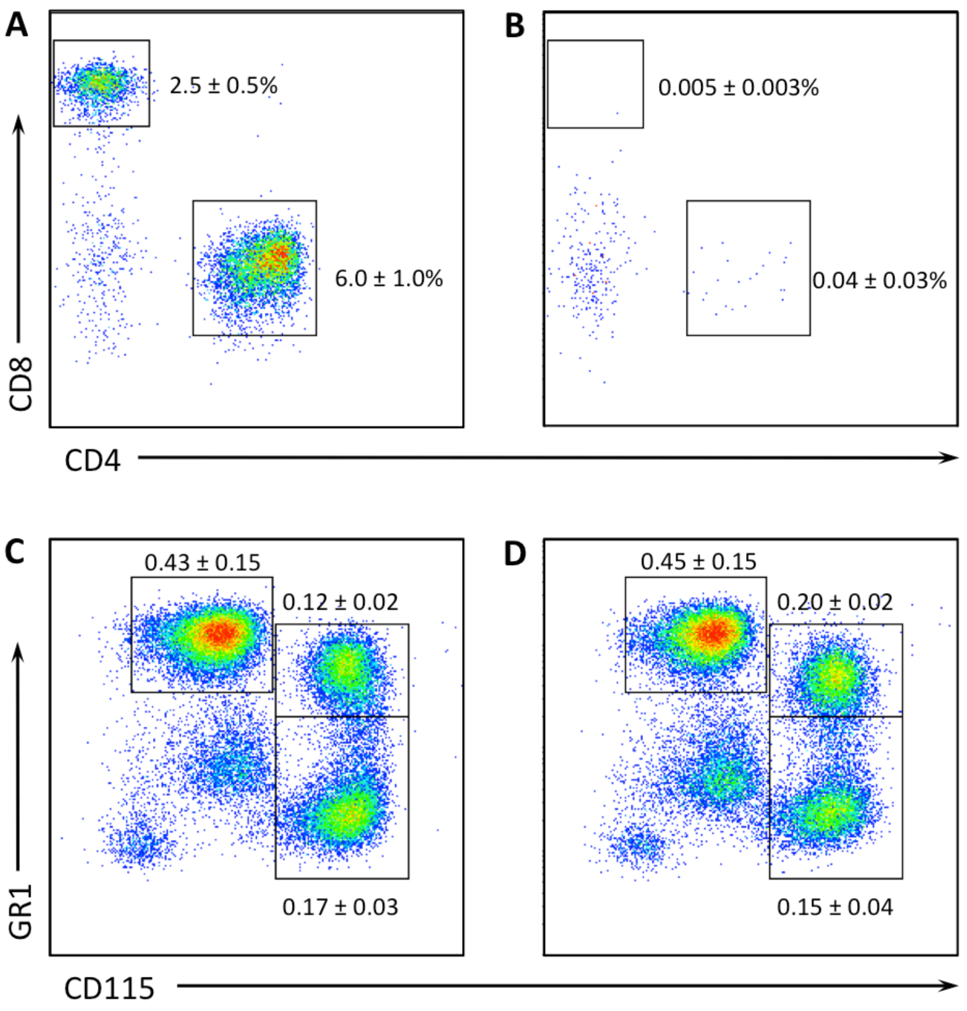

Supplemental figure 3. Antibody-mediated depletion of CD4$^+$ and CD8$^+$ T lymphocytes

Representative dot plots of circulating CD3$^+$ lymphocytes (A, B) and CD11b$^+$ myeloid cells (C, D) in the blood of an ApoE$^{-/-}$ mouse on HFD before (A, C) and 7 days after (B, D) administration of an i.p. dose of 100μg anti-CD4 (YTA 3.1) and 200μg anti-CD8 (YTS 169) depleting antibodies. (A, B) CD4$^+$ and CD8$^+$ T lymphocytes were quantified as percentage of the leukocyte gate (mean ± SD for n=7 mice). (C, D) CD115$^-$GR1^{hi} neutrophils; CD115$^+$GR1^{hi} and CD115$^+$GR1^{int+lo} monocytes, presented as million cells/mL (mean ± SD for n=7 mice).

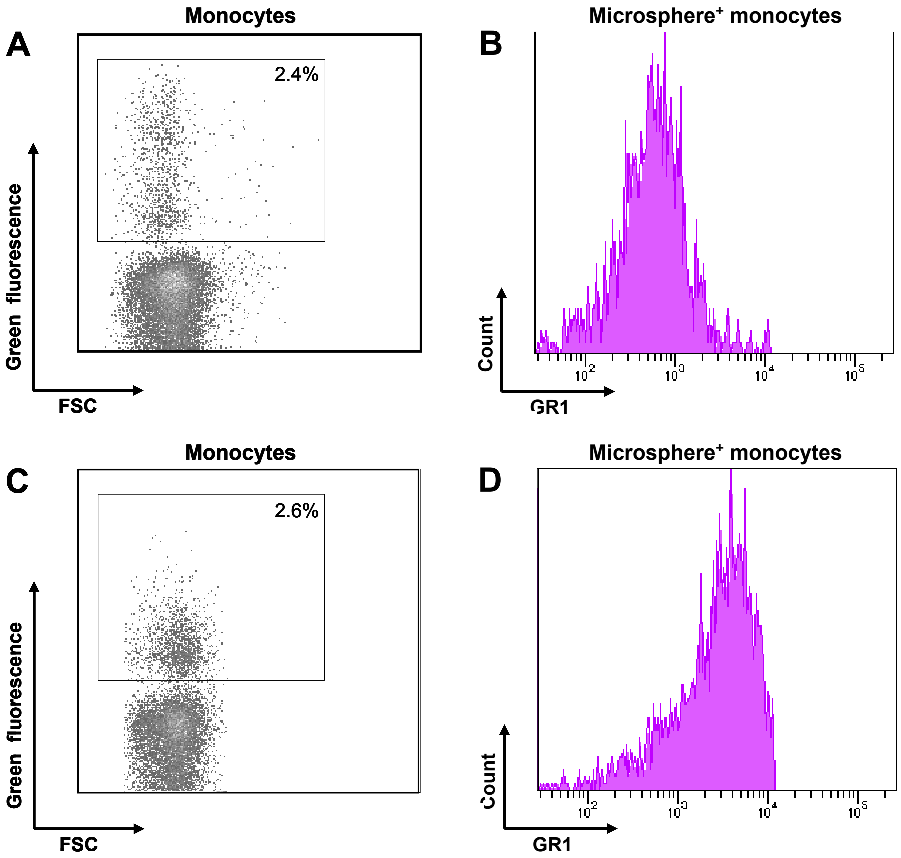

Supplemental figure 4. ***In vivo*** **monocyte labelling**

Fluorescent cytometry analysis of CD11b$^+$CD115$^+$ blood monocytes 3 days following *in vivo* labelling of either resident Ly-6C^{lo} (GR1^{lo}) (A, B) or inflammatory Ly-6C^{hi} (GR1^{hi}) (C, D) monocytes in ApoE$^{-/-}$ mice using green fluorescent latex microspheres. (A, C) Percentage of microsphere$^+$ monocytes of total monocytes. (B, D) GR1 expression on the microsphere$^+$ monocytes gated in (A) and (C) respectively. The Ly-6C^{lo} monocytes were labelled by direct i.v. injection of fluorescent microspheres. The Ly-6C^{hi} monocytes were labelled by initial monocyte depletion with clodronate-loaded liposomes injected i.v. followed by i.v. microsphere injection 18 hours later as described in methods.

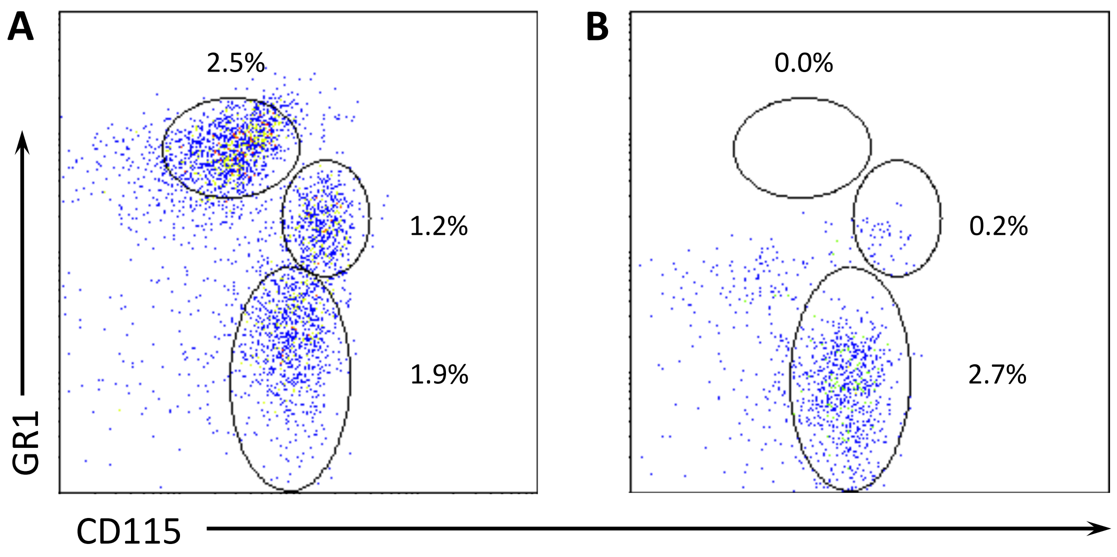

Supplemental figure 5. Anti-GR1 antibody-mediated depletion of circulating neutrophils and Ly-6C^{hi} (GR1^{hi}) monocytes

Representative dot plots of circulating CD115^{-}GR1^{hi} neutrophils, CD115^{+}Ly-6C^{hi} (GR1^{hi}) and CD115^{+}Ly-6C^{int+lo} (GR1^{int+lo}) monocytes gated as CD11b^{+} cells in the blood of a C57BL/6 mouse before (A) and after (B) administration of 2 consecutive i.p. doses of 200μg anti-GR1 (RB6-8C5) depleting antibody. The mouse was sacrificed 2 days after the second antibody injection. The cells are quantified as percentage of the leukocyte gate. The plots are representative for n=5 mice.
